# Gene–Environment Interplay in Adolescent Drinking Behavior

**Published:** 2004

**Authors:** Richard J. Rose, Danielle M. Dick

**Affiliations:** Richard J. Rose, Ph.D., is a professor emeritus in the Department of Psychology, Indiana University, Bloomington, Indiana. Danielle M. Dick, Ph.D., is an assistant professor in the Departments of Psychiatry and Psychology, Washington University, St. Louis, Missouri

**Keywords:** adolescent, young adult, age of onset, AOD (alcohol and other drug) use initiation, AOD use pattern, genetic theory of AODU (alcohol and other drug use), hereditary factors, environmental factors, hereditary vs. environmental factors, social environmental risk and protective factors, family risk and protective factors, longitudinal study, twin study, monozygotic twin, dizygotic twin, the *FinnTwin* studies, Finland, Collaborative Study on the Genetics of Alcoholism (COGA)

## Abstract

Many people begin to consume alcohol and establish drinking patterns during adolescence, making this an important developmental period for alcohol researchers to study. Both drinking initiation and establishment of drinking patterns are influenced to varying degrees by genetic as well as environmental factors. Using twin studies conducted in Finland and other countries, researchers have analyzed the specific genetic and environmental influences as well as the gene–environment interactions that shape drinking behavior in adolescence. These studies indicate that drinking initiation is determined primarily by environmental influences, whereas the establishment of drinking patterns is determined mostly by genetic factors, which themselves are subject to moderation by the environment.

The years between early adolescence and young adulthood are a crucial period for alcohol researchers to study, because drinking typically is initiated during adolescence, and by young adulthood, individual differences in established drinking patterns are evident. The earliest stages of alcohol use merit particular attention because research has suggested that the initiation of alcohol use must be distinguished from the frequency, quantity, and density (i.e., the number of drinks consumed in a certain length of time) of alcohol use once drinking is initiated. Consequently, analyses in adults with already established drinking patterns can explain only part of the mechanisms and contributing factors underlying the development of alcohol abuse and dependence. To obtain a more comprehensive explanation of these processes, it also is necessary to study the factors influencing drinking initiation.

Another reason to focus on early adolescent drinking comes from research evidence indicating that people who begin drinking at an earlier age have a heightened risk for negative consequences of drinking, such as alcohol abuse or alcohol dependence. For example, interviews of adults consistently and convincingly have shown a strong association between an early initiation of drinking and later alcohol-related problems: People who report drinking as early as age 13 or 14 are much more likely to exhibit alcohol-related behavior problems in adulthood than people who began drinking at a later age (Grant and Dawson 1997). The association of drinking problems with earlier age of initiation is particularly strong in people with a family history of alcoholism, but also exists in people without a family history.

But what is the relevance of this association? In most cases, it is hazardous to draw a causal connection based on such observations. Thus, it is possible that drinking initiation at an earlier age is causally related to greater vulnerability to alcohol’s cumulative effects. Alternatively, early onset of drinking and a greater likelihood of later problems may simply be correlated—that is, they are independent signs or symptoms of people who are at elevated risk of alcohol dependence. In the latter case, prevention efforts designed to promote delays in age of drinking initiation would do little to reduce the prevalence of adult alcoholism—an issue that is important to resolve. One approach to doing this is to conduct longitudinal studies that follow, over several years, genetically informative samples, such as samples of twins who differ in their age at onset of drinking.

This article reviews recent findings from two ongoing longitudinal twin studies conducted in Finland that elucidate the contributions of genetic and environmental influences to initiation of drinking and later trajectories of alcohol use and abuse. Data from these population-based *FinnTwin* studies help investigators address questions about factors contributing to various stages of alcohol use. The resulting research has underscored the importance of the concept of dynamic gene–environment interactions.

## The General Role of Genetic and Environmental Factors in Alcohol-Related Behavior

Both genetic and environmental factors contribute to the different stages in a person’s drinking history. The factors that influence individual differences in whether, and at what age, drinking is initiated are different in kind from the factors that influence individual variations in patterns of drinking, once the acute subjective effects of alcohol ingestion are experienced. Moreover, it is clear from numerous studies that although genetic factors have some influence on drinking behavior, the effects of genetic differences between people can be enhanced or reduced by variations in the environments in which the people live—that is, there are gene–environment interactions.

A person’s susceptibility to developing alcohol dependence and other alcohol-related problems manifests as an unfolding developmental process that involves both genetic and environmental factors and is continuously modulated by gene–environment interplay. This concept of a complex developmental process is supported by fundamental findings in recent behavior-genetic research that have profoundly altered the earlier view that people are passive players who mechanically mirror their environmental experience. Instead, recent research findings indicate the existence of extensive gene–environment correlations, whereby people are active agents who help create the experiences to which they are exposed, selectively seek environments to match their specific dispositions, and modify their environments through their actions. In short, new research suggests that constant gene-by-environment interplay is involved in the development of alcoholism risk over time.

To understand the factors affecting the development of alcoholism, it is essential to appreciate this continuing interaction of genetic and environmental influences (e.g., how a child’s behavioral dispositions interact with that child’s household environment). Similarly, it is important to acknowledge that the household environment does not stand alone, but is “nested” within neighborhoods, schools, communities, and cultures. Accordingly, the ways in which a child expresses his or her internal dispositions are modulated by the adult models, parenting practices, peer interactions, access to alcohol, and school and neighborhood environments to which the child is exposed. All of these factors either enhance or reduce the risk for alcoholism that results from the child’s genetic makeup.

## The *F****INN****T****WIN*** Studies

Longitudinal studies using genetically informative samples enable researchers to assess the magnitude of genetic and environmental influences and their interplay across development for a variety of behaviors. Therefore, these research designs also are particularly useful in understanding the development of drinking behaviors. The most widely used design is a longitudinal/developmental study of child or adolescent twins and their parents. Several research groups in the United States and other countries are engaged in developmental behavior-genetic research of this kind, and many of these groups explore risk factors for alcoholism as part of their research aims. This article focuses on two longitudinal studies of young Finnish twins and their parents known as the *FinnTwin* studies.

Finland is particularly suitable for conducting longitudinal twin studies because of the availability of comprehensive birth records, the ease of locating twins as they grow up, and the high level of compliance of organizations and individuals with research requests.

To acquire a large sample of twins that is representative of the entire population, it is almost imperative that investigators have access to a comprehensive birth registry for the region or country under investigation. However, many countries, including the United States, do not have such registries. Some researchers in the United States have attempted to obtain comprehensive data sets by identifying all twin students enrolled in a large public school system or by systematically accessing birth records within a State and then searching for the current addresses of the twins so identified.

In the Nordic countries, in contrast, the entire population is enrolled at birth into a centralized population registry. In Finland, every newborn citizen is given a unique personal identification number that includes the person’s date of birth and a link to the biological mother. Accordingly, researchers who have approval to access the registry can identify all people born on the same day to the same mother, thereby identifying the nation’s entire population of twins and other multiples. Moreover, Finnish law requires all citizens to report all residential moves in a timely manner, which means that families of twins can be readily located and, in principle, followed up without loss. Finally, for cultural and historic reasons, compliance with requests for research participation is unusually high in the Nordic countries. This high compliance rate and the requirement to report residential moves make it possible to conduct followup assessments with large numbers of subjects, which otherwise presents a particular challenge when researching drinking behavior and its consequences (Kaprio et al. 2002).

### Design of the *FinnTwin* Studies

The two *FinnTwin* studies differ in the age groups they study and in their followup intervals. One study, *Finn-Twin16*–*25*, includes all twins born in five consecutive birth cohorts, 1975 through 1979, who were enrolled in the study when they reached age 16. Systematic followup surveys of these twins were completed at ages 17 and 18.5 and at one point when the twins were between ages 22 and 25. The second study, *FinnTwin12*–*17*, identified all twins in the five consecutive birth cohorts 1983 through 1987 who were enrolled at age 11 or 12, with followup assessments at ages 14 and 17.

Each *FinnTwin* study includes about 2,500 pairs of twins, with equal proportions of brother–sister, brother–brother, and sister–sister twin pairs. Among the same-sex pairs, about half are identical, or monozygotic (MZ) twins and half are fraternal, or dizygotic (DZ) twins. MZ twins develop when a single egg is fertilized by a single sperm, and the resulting embryo divides shortly after fertilization to produce two individuals who share all their genes. DZ twins, in contrast, develop when two egg cells are independently fertilized by separate sperm at about the same time (or in close temporal proximity), resulting in the development of two individuals who, like ordinary siblings, share, on average, half of their genes.

The fundamental approach in twin study research is to compare the extent to which MZ twins resemble each other in relation to a specific trait or behavior with the resemblance between DZ twins for the same trait or behavior. In MZ twins, the genetic influences on that trait or behavior are identical because the co-twins share all their genes. In DZ twins, however, the genetic influences can differ because the co-twins share only about 50 percent of their genes. With respect to environmental influences, there should be no substantial differences between MZ and DZ twins because, in childhood and adolescence, all or nearly all twin pairs live with one another and with the same biological parent or parents. Therefore, both MZ and DZ twins will share equally important aspects of their household experience, peers, neighborhood, school, and other environmental factors that may enhance or reduce the risk for early alcohol use and abuse.

Based on these premises regarding genetic and environmental influences, the following expectations can be articulated:

- If genetic factors are primarily important in the development of a measured trait or behavior, MZ co-twins will resemble one another about twice as much as do DZ co-twins.
- If genetic factors are of only modest importance and shared environmental influences are predominant, the two kinds of twins will show comparable resemblance for the trait or behavior studied, because both share the environmental influences relevant to that outcome.

The following sections summarize some of the findings of the *FinnTwin* studies regarding the contributions of various genetic and environmental factors to a variety of drinking-related behaviors. In general, the results from the *FinnTwin* studies have been consistent with ongoing research with adolescent twins in other European countries as well as in the United States.

## The Initiation of Alcohol Use

Twin studies consistently have demonstrated that initiation of alcohol use is largely influenced by environmental rather than genetic factors (Hopfer et al. 2003), a conclusion that is supported by the results from *FinnTwin12*–*17*, which addressed this issue. Nearly two-thirds (64 percent) of the twins in this study reported never having used alcohol at age 14, whereas 36 percent reported that they had begun drinking alcohol at this age. Comparisons of the MZ and DZ twin pairs in the study determined that the environmental factors shared by co-twins played the largest role in influencing abstinence/drinking at age 14. These factors—which included the familial environment and the nonfamilial environments of peers, schools, and neighborhoods—accounted for 76 percent of the variation in drinking initiation in both boys and girls (Rose et al. 2001*b*).

One relevant question in this context is whether the same factors influence drinking initiation in 14-year-old boys and girls. Although the *magnitude* of importance of environmental factors is the same in boys and girls, it is possible that *different* environmental factors may be acting in each gender. Twin studies allow researchers to estimate the extent to which the same or different environmental factors influence boys and girls. In the *FinnTwin12*–*17* study, 77 percent of the environmental factors that contribute to the twin siblings’ similarity in age of drinking initiation are the same for adolescent males and females (i.e., the correlation between environmental factors was estimated at 0.77). Thus, although many environmental influences impacting drinking initiation are the same for girls and boys, some unidentified environmental influences are specific to each gender.

Although the *FinnTwin* data provided little evidence of genetic effects on drinking initiation by age 14, some gender differences were apparent in that respect as well. Genetic effects were found to have a modest influence on girls (accounting for 18 percent of the variance in girls’ drinking at age 14) but no influence on boys’ drinking at the same age. Perhaps this difference reflects the fact that girls are more developmentally mature at age 14 than boys. By this age, most girls already have undergone significant pubertal development, whereas most boys are just beginning to experience pubertal changes. Girls’ more advanced maturation may foster associations with older friends and peers that offer greater access to alcohol, thereby creating opportunities for girls to express drinking-related genetic predispositions at an earlier age.

## Symptoms of Alcohol Dependence at Age 14

It is uncommon for 14-year-olds to exhibit symptoms of alcohol dependence, and at this age such symptoms are not attributable to genetic effects. To explore the issue of alcohol dependence at this early age in more detail, researchers administered a structured face-to-face interview to a sample of more than 1,800 twins from the *FinnTwin 12*–*17* study. Only 12.4 percent of the 14-year-old twins in this sample reported any symptoms of alcohol dependence, and only 1 percent met the criteria for a diagnosis of alcohol dependence. Moreover, more girls than boys reported alcohol dependence symptoms or met diagnostic criteria; this gender difference may be attributable to the fact that, at this age, girls are more likely than boys to be drinking.

For comparison, the investigators also determined the frequency of symptoms of conduct disorder (CD) in the same sample of twins. This analysis yielded several results that differed sharply from those obtained regarding alcohol dependence symptoms (Rose et al. 2004):

- Symptoms of CD were common among the adolescent twins: 44 percent of the sample reported one or more symptoms of CD, and 12 percent met diagnostic criteria for the condition.
- CD symptoms were more common among boys, who accounted for 65 percent of all twins diagnosed with CD.
- Genetic factors played a much different role in CD than in alcohol dependence. Whereas genetic factors had a negligible influence on alcohol dependence, they had a substantial influence on symptoms of CD.

Despite these differences, the two sets of symptoms are significantly correlated (0.50), meaning that the CD symptoms were more common among twins with a diagnosis of alcohol dependence and, similarly, the symptoms of alcohol dependence were more common among twins diagnosed with CD. However, because the twin data produced no evidence that genetic factors contribute to alcohol dependence symptoms at this age, the association of alcohol symptoms with CD symptoms must be attributed entirely to environmental factors that influence both conditions (Rose et al. 2004). One inference from these results is that CD is an early manifestation of a genetic predisposition that later contributes to the development of alcohol-related problems and alcohol dependence. Therefore, efforts to identify adolescents at risk for developing alcoholism and to create opportunities for targeted interventions should focus on identifying adolescents exhibiting symptoms of CD. Other research designs, such as studies of adopted children and their biological and adoptive families, also may yield important information on the genetic relationship between CD and alcohol dependence.

## Identifying Specific Environmental Influences on Drinking

The environmental factors shared by siblings, and assessed in twin studies such as *FinnTwin*, include not only household environmental characteristics (such as family structure and status, parenting practices, and home atmosphere) but also environmental influences exerted through schools, neighborhoods, and communities. For some behaviors and at some ages, the nonfamilial experiences that children share with their peers at school and in their neighborhoods may be more salient than the household influences they share with their families in their homes. Drinking in early adolescence may be one such behavior. To evaluate this hypothesis, researchers can assess the contribution of nonfamilial environments to children’s behavior by comparing the resemblance of unrelated classmates of twin siblings to one another and to their classmate twins.

The *FinnTwin12*–*17* study undertook such an approach (Rose et al. 2003). For this analysis, researchers linked each twin with a classmate control subject of the same gender who had the same year of birth, lived in the same neighborhood, and attended the same school and classroom as the twin.

As discussed earlier, behavioral resemblance of co-twins results from their shared genetic makeup and from the household experiences they share with each other and with their parents, as well as experiences they share with peers outside the home. In contrast, any behavioral resemblance between the twins and their classmates (who are genetic strangers and share no household experiences with the twins being studied) must result entirely from experiences shared in school and neighborhood. Accordingly, this research approach provides a direct estimate of the role of environmental influences associated with schools and neighborhoods.

In the *FinnTwin12*–*17* study, behavior of the twins and the classmate control subjects was assessed when the children were 11 or 12 years old by means of a questionnaire administered during school hours under research supervision. The questionnaire included three drinking-related items illustrating nonfamilial environmental effects:

- “Have you ever drunk alcohol?”
- “Have you ever seen any of your friends drunk?”
- “Have you ever drunk alcohol with your friends without adults around?”

The prevalence of affirmative answers to these questions differed widely, as would be expected among children at this age. For example, more than two-thirds of the boys and more than half of the girls admitted to having had a drink of alcohol—which is not uncommon in Finnish family culture, where children may have a drink at holiday time and with parental knowledge. In contrast, only 18 percent of boys and 11 percent of girls reported having seen friends drunk, and only 7 percent of the boys and girls reported ever having drunk alcohol with their friends without adults around.

**Figure 1:**
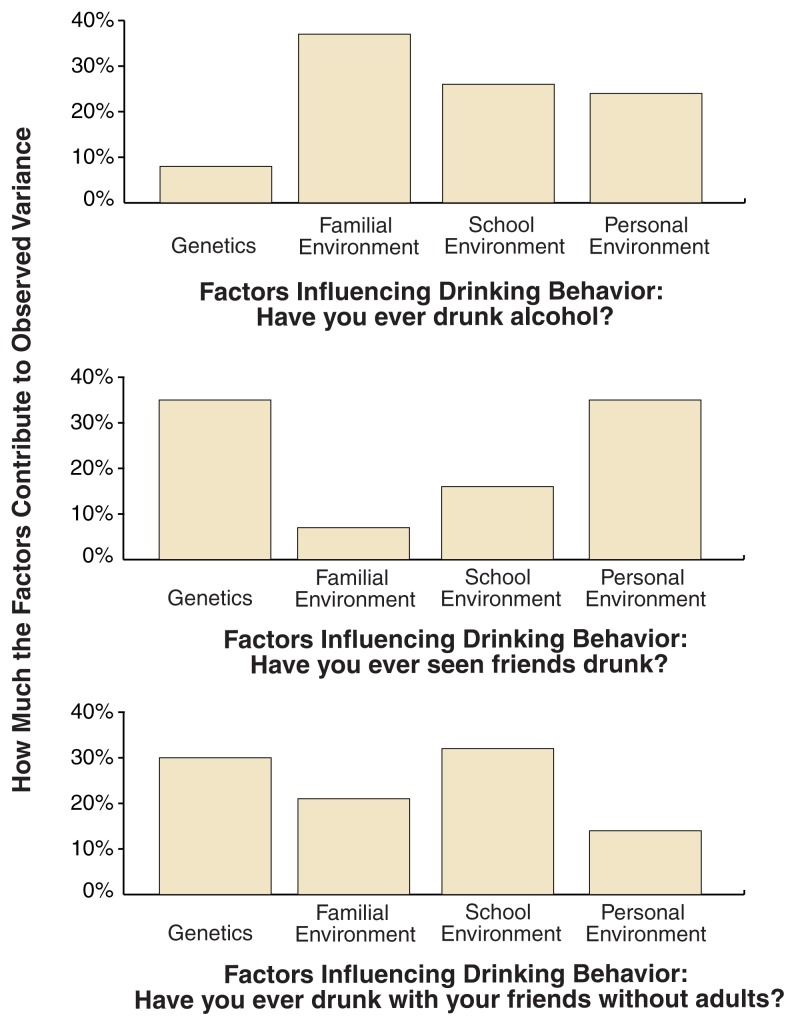
The relative contributions of genetic factors, familial environment, school environment, and personal environment to drinking-related experiences of Finnish twins ages 11 and 12. Influences from the familial environment are shared by twin siblings but not shared with their unrelated classmates; school-based neighborhood effects are shared fully by twin siblings and their classmates. Data were obtained from 631 same-sex twin pairs (i.e., 1,262 twins) and a matched, same-sex classroom control subject for each twin. SOURCE: Rose et al. 2003.

The investigators determined the relative influences of genetic and specific environmental factors on these three variables based on the relative similarities of MZ and DZ twins and their classmate control subjects (see figure 1). They found that genetic effects accounted for only 8 percent of the individual differences in whether the children had begun to use alcohol, but they accounted for 30 percent of the variance in alcohol use without adult supervision (Rose et al. 2003). Conversely, familial effects were very influential in initiation of use, accounting for nearly 40 percent of the variance. They had little influence, however, on whether these children had ever seen any of their friends drunk, which was more closely linked to genetic influences and the effects of the child’s personal experiences (i.e., experiences not shared with the twin sibling). Finally, experiences shared with school classmates contributed significantly to each of these alcohol-related experiences.

Thus, this expanded twin–classmate control design has identified important influences on drinking behavior that originate both within and outside the family environment. These findings document the varying influences of a person’s genetic dispositions on different aspects of drinking behavior in early adolescence.

## Drinking Patterns Across Adolescence

The relative contributions of genetic and environmental factors to the drinking behavior of young people change during adolescence. As previously described, familial and environmental influences predominate in their impact on initiation of drinking behavior. When people move from early experimental alcohol use to more established patterns of use, however, the importance of individual genetic predispositions increases greatly, and the importance of common environmental factors decreases accordingly. Findings from the *FinnTwin 16*–*25* study illustrate this process.

One component of *FinnTwin16*–*25* was an assessment of the twins at ages 16, 17, and 18.5. Over this 30-month period of followup, the number of abstinent participants decreased dramatically, from about 25 percent of twins at age 16 to only about 10 percent of twins at age 18.5. Conversely, the proportion of twins who reported drinking at least twice monthly more than doubled over the 30-month period, and at age 18.5 more than 60 percent of twin boys and girls drank that often (Rose et al. 2001*a*).

**Figure 2:**
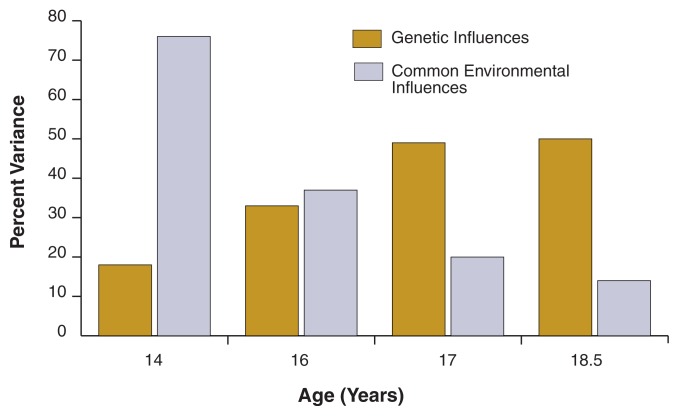
The changing influence of genetic and common environmental factors on drinking behavior during adolescence. Data for age 14 illustrate influences on initiation of drinking and were taken from the *FinnTwin12*–*17* study. Data for all other ages illustrate influences on drinking frequency and were taken from the *FinnTwin16*–*25* study. Genetic influences at age 14 are significant only for girls; boys show no evidence of genetic influences on drinking initiation at this age. SOURCE: Rose et al. 2001a,b.

Interestingly, the influence of genetic factors also increased across this age range. Genetic factors accounted for only 33 percent of the variation in drinking frequency at age 16, but 50 percent of the variation by age 18 (Rose et al. 2001*a*). At the same time, common environmental factors became less important, accounting for 37 percent of the variance at age 16, but only 14 percent by age 18 (see figure 2). This shift in the relative influence of environmental factors is even more dramatic when one includes the data on drinking initiation from 14-year-old twins in the *FinnTwin12*–*17* study. Taken together, these findings indicate that familial and community factors appear to have their greatest impact on adolescents’ decisions to initiate alcohol use and on their early experimental drinking; as drinking patterns develop, differentiate, and stabilize across adolescence, however, genetic influences on drinking patterns become increasingly important.

## Gene–Environment Interaction

Although the contribution of genetic influences to alcohol use increases during adolescence, environmental factors do not become irrelevant but play an important role in moderating the influence of genetic predispositions. For example, findings from the *FinnTwin* studies demonstrated that urban or rural residency has a significant impact on the relative contributions of genetic and other environmental factors which affect adolescent drinking. Genetic influences on drinking frequency were much stronger among twins living in urban settings, where they accounted for 34 percent of the variance in drinking at age 16, than among twins residing in rural Finland, where they accounted for 18 percent of the variance in drinking (Rose et al. 2001*a*). Conversely, common environmental effects accounted for more than 50 percent of the variation in drinking patterns among rural youth, but for only one-third of the variance in youth living in urban settings. This finding has been replicated recently in a twin study conducted in Minnesota (Legrand et al. 2003).

Subsequent analyses have attempted to identify the processes underlying this phenomenon. What characteristics of urban settings allow for greater expression of genetic effects? Why do environmental influences exert a larger influence in rural settings? To explore these questions, researchers have expanded the classic twin model to incorporate more continuous measures of the environment and examine a series of socioregional variables that they hypothesized to be more closely related to the interaction effect (Dick et al. 2001).

Finland offers an opportunity to explore such questions because the country is divided into municipalities—local government units that levy taxes and assume responsibility for education, health care, and social services. For these municipalities, whether in the metropolitan Helsinki area or in sparsely populated areas of Finnish Lapland and the Åland Islands, a wealth of sociodemographic information is available that may be relevant to risk for adolescent drinking.

To study the influence of community-level factors on adolescent drinking outcomes, Dick and colleagues (2001) linked this sociodemographic information to each twin pair’s residency code. By doing so, the investigators were able to identify characteristics of the communities in which each twin pair lived that could be related to risk. The specific sociodemographic variables assessed to characterize the urban/rural interaction effect included the following:

- Percentage of young adults, ages 20–24, in a municipality.
- Neighborhood instability, as indicated by the percentage of migration into and out of a given region.
- Per capita expenditure on alcohol in each region, relative to the mean amount spent in the entire country.

The researchers found that each of these variables had dramatic effects on the genetic and environmental influences on drinking behavior, with a more than fivefold difference in the magnitude of genetic effects between environmental extremes. Thus, communities characterized by more young adult role models, greater social mobility, and higher alcohol sales allowed for increased expression of genetic dispositions that contribute to individual differences in adolescent drinking. On the other hand, communities with more social stability created an environment in which common environmental effects, within families and within communities, assumed greater importance.

Analyses of other twin data sets also have demonstrated the importance of environmental factors in moderating genetic and environmental influences on drinking patterns. In Australian twin data, marital status moderated the importance of genetic effects on alcohol consumption in women (Heath et al. 1989)—that is, living in a marriage-like relationship reduced the impact of genetic influences on drinking. In this study, genetic factors accounted for only half as much variance in drinking among married women (31 percent) as among unmarried women (60 percent) (Heath et al. 1989).

In another study, on the effect of religiosity on alcohol use among women, genetic factors played a significant role in women who had not had a religious upbringing, dwarfing the influence of shared environment. By contrast, there was no evidence of genetic influence in women with a religious upbringing, but shared environment had a substantial impact (Koopmans et al. 1999).

Lastly, researchers recently have begun to explore the potential moderating role of parenting factors on adolescent alcohol use. Preliminary analyses have provided strong evidence that changes in the relevance of genetic and environmental factors are related to differences in parental monitoring, involvement, and support (Dick et al. 2005).

Complementary twin designs, such as studies of the offspring of adult twins, also provide rich opportunities to study gene–environment interaction and the potential moderating role of environmental factors, such as parenting practices and parental models (D’Onofrio et al. 2003).

## Molecular Genetic Advances in Understanding Drinking Patterns

Advances in genetic analysis and in the technology used to determine a person’s genetic makeup (i.e., genotype) now make it possible to move beyond estimates of unmeasured (i.e., latent) sources of genetic variance. Researchers have begun to identify specific genes involved in complex behavioral outcomes such as alcohol use.

Using a candidate gene approach, which examines the influence of specific genes suspected of playing a role in the behavior under investigation, two studies recently reported associations between specific gene variants and drinking behavior among college students. The first study investigated the role of variation in the gene encoding a protein called the serotonin transporter. This study found that in a sample of White students, those with a particular version of this gene engaged in binge drinking more often, drank to intoxication more often, and consumed more alcoholic drinks per drinking occasion than students with other variants of the gene (Herman et al. 2003).

Another study focused on the gene that encodes aldehyde dehydrogenase (ALDH), an enzyme involved in the breakdown of alcohol in the body. This study reported that Asian American college students who carried a particular version of the ALDH gene which results in less efficient alcohol breakdown were less likely to be regular drinkers and engage in binge-drinking episodes; they also reported a lower number of maximum drinks consumed in a 24-hour period than Asian students with other ALDH variants (Wall et al. 2001).

Such studies are complemented by large-scale efforts to identify genes that contribute to alcoholism. One of these efforts, funded by the National Institute on Alcohol Abuse and Alcoholism, is the Collaborative Study on the Genetics of Alcoholism (COGA). COGA researchers recently published reports of several genes associated with alcohol dependence in adults (Edenberg et al. 2004; Dick et al. 2004*a*; Wang et al. 2004), and some of these findings already have been replicated by other investigators (Covault et al. 2004; Lappalainen et al. 2005; Luo et al. 2005).

The next challenge will be to expand these findings to children and adolescents to better understand how specific predisposing genes influence trajectories of drinking and other behavioral problems across development. The identification of specific genes influencing alcohol use and abuse will enable researchers to begin to decipher how the effects of these genes are modified by specific environmental influences. Elucidating the influence of specific genes on various aspects of drinking, the actions of these genes, and their interactions with specific environmental risk factors should dramatically enhance our understanding of the development of alcohol use and abuse.

## Conclusions and Future Outlook

Both genetic and environmental factors contribute to the development of patterns of alcohol use during adolescence. The relative importance of genetic and environmental factors, however, varies for different phases of alcohol use and different stages of development. Environmental influences originating both within the family and in the child’s school and neighborhood greatly influence the initiation of alcohol use. Once drinking has been initiated, however, genetic influences assume increasing importance across development, as individual differences in patterns of drinking emerge. The degree to which genetic effects contribute to adolescent drinking patterns also is subject to moderation by the environment, with factors such as religiosity, marital status, and regional residency all contributing to the relative importance of genetic and environmental effects on alcohol use.

Other recent efforts have been directed toward identifying specific genes contributing to variation in drinking patterns. As scientists begin to understand more about the nature of gene–environment interactions, molecular genetic studies can be designed that take this emerging information into account (Kendler and Eaves 1986). The potential of the molecular genetics approach has been illustrated by a recent study that found an association between a variant of the serotonin transporter gene and major depression, but only in people who had experienced stressful life events (Caspi et al. 2003). Large-scale collaborative projects have begun to identify genes involved in alcohol dependence and related behaviors (Dick et al. 2004*a*; Edenberg et al. 2004; Wang et al. 2004).

Once researchers have identified specific genes associated with complex disorders such as alcoholism, they can begin to address questions regarding developmental effects or gene–environment interactions associated with these genes (Dick et al. 2004*b*). Such questions include whether genetic variants affect different behaviors at different developmental stages, whether particular genetic variants are more or less important at various ages, or whether the risk associated with a specific gene varies according to environmental context. Integrating the knowledge obtained from behavior-genetic studies with findings emerging from the areas of statistical and molecular genetics promises to enhance our understanding of the factors that contribute to drinking among adolescents and young adults.
